# Trained Immunity Confers Prolonged Protection From Listeriosis

**DOI:** 10.3389/fimmu.2021.723393

**Published:** 2021-09-17

**Authors:** Charlotte Théroude, Marta Reverte, Tytti Heinonen, Eleonora Ciarlo, Irene T. Schrijver, Nikolaos Antonakos, Nicolas Maillard, Florian Pralong, Didier Le Roy, Thierry Roger

**Affiliations:** Infectious Diseases Service, Department of Medicine, Lausanne University Hospital and University of Lausanne, Epalinges, Switzerland

**Keywords:** trained immunity, innate immunity, infection, sepsis, listeria, neutrophils, myelopoiesis, immunometabolism

## Abstract

Trained immunity refers to the ability of the innate immune system exposed to a first challenge to provide an enhanced response to a secondary homologous or heterologous challenge. We reported that training induced with β-glucan one week before infection confers protection against a broad-spectrum of lethal bacterial infections. Whether this protection persists over time is unknown. To tackle this question, we analyzed the immune status and the response to *Listeria monocytogenes* (*L. monocytogenes*) of mice trained 9 weeks before analysis. The induction of trained immunity increased bone marrow myelopoiesis and blood counts of Ly6C^high^ inflammatory monocytes and polymorphonuclear neutrophils (PMNs). *Ex vivo*, whole blood, PMNs and monocytes from trained mice produced increased levels of cytokines in response to microbial products and limited the growth of *L. monocytogenes*. *In vivo*, following challenge with *L. monocytogenes*, peripheral blood leukocytes were massively depleted in control mice but largely preserved in trained mice. PMNs were reduced also in the spleen from control mice, and increased in the spleen of trained mice. In transwell experiments, PMNs from trained mice showed increased spontaneous migration and CXCL2/MIP2α-induced chemotaxis, suggesting that training promotes the migration of PMNs in peripheral organs targeted by *L. monocytogenes*. Trained PMNs and monocytes had higher glycolytic activity and mitochondrial respiration than control cells when exposed to *L. monocytogenes*. Bacterial burden and dissemination in blood, spleen and liver as well as systemic cytokines and inflammation (multiplex bead assay and bioluminescence imaging) were reduced in trained mice. In full agreement with these results, mice trained 9 weeks before infection were powerfully protected from lethal listeriosis. Altogether, these data suggest that training increases the generation and the antimicrobial activity of PMNs and monocytes, which may confer prolonged protection from lethal bacterial infection.

## Introduction

Innate immune cells express pattern-recognition receptors (PRRs) involved in the sensing of microbial-associated molecular patterns (MAMPs) and damage-associated molecular patterns (DAMPs) released by injured or stressed cells. The main families of PRRs are Toll-like receptors (TLRs), NOD-like receptors (NLRs), c-type lectins receptors (CLRs), RNA sensors (RIG-I-like receptors and DExD/H-box helicases) and cytosolic DNA sensors (CDSs) (1, 2). The interaction of PRRs with MAMPs/DAMPs triggers intracellular signaling pathways among which nuclear factor-κB (NF-κB), mitogen-activated protein kinases (MAPKs) and interferon (IFN) response factor (IRF) signaling pathways. Innate immune cells shift their metabolism to meet the energy demand necessary for defense functions. These processes are associated with epigenetic changes that modulate gene expression and the effector functions of innate immune cells.

The restriction of immunological memory to the adaptive immune system has been challenged by the observations of systemic acquired resistance in plants and nonspecific effects of vaccines and *Candida albicans* infection on host defenses in vertebrates (3, 4). The concept of trained immunity was proposed to account for innate immune memory in mammals (5). Recent consensus definitions restricts trained immunity to characterize “faster and greater response against a secondary challenge with homologous or even heterologous pathogens” (6). Trained immunity induced by MAMPs/DAMPs has been studied principally using the fungal cell wall component β-glucan, *Candida albicans* and the Bacillus Calmette-Guérin (BCG). The impact of trained immunity is most often assessed one week after induction of training. Yet, BCG-induced training has been detected in PBMCs up to 3 months after vaccination of humans, and in alveolar macrophages up to 16 weeks after challenge of mice (7–9). Remarkably, innate immunological imprinting persisted 6 months in brain-resident macrophages of mice challenged with LPS (10).

The molecular mechanisms underlying trained immunity include metabolic, epigenetic and functional reprogramming of cells. These adaptations were initially described in monocytes and macrophages, and later reported in hematopoietic stem cells (HSCs), non-immune cells and stem cells including skin and lung epithelial stem cells (7, 10–18). Epigenetic changes affecting DNA and chromatin in trained monocytes/macrophages promote their metabolic rewiring and expression of proinflammatory genes.

Contrary to adaptive immune memory, innate immune memory conferred by trained immunity is not antigen specific. This goes along with human epidemiological studies reporting strong non-specific effects on host defenses of live vaccines (BCG, polio, smallpox, measles), and proof of principle studies showing that BCG vaccination protects from controlled infection by yellow fever and malaria (19–22). Further demonstrating the broad effects of trained immunity, we reported that trained immunity conferred protection from clinically relevant pathogens inoculated through diverse routes to induce peritonitis, systemic infections, enteritis and pneumonia (23).

Many aspects of trained immunity remain to be addressed (24, 25). Among others, the length of protection conferred by trained immunity against lethal acute infections is unknown. In the present study we questioned whether trained immunity induced persistent changes to protect from acute listeriosis. We show that myelopoiesis was increased 9 weeks after the induction of training, affecting peripheral blood leukocyte pool and functions. Moreover, mice trained 9 weeks prior to infection were remarkably protected from lethal listeriosis.

## Materials and Methods

### Ethics Statement

Animal experiments were approved by the Service des Affaires Vétérinaires, Direction Générale de l’Agriculture, de la Viticulture et des Affaires Vétérinaires (DGAV), état de Vaud (Epalinges, Switzerland) under authorizations n° 876.8/9 and 877.9/10 and performed according to Swiss and ARRIVE guidelines and according to Directive 2010/63/EU of European Union.

### Mice and Cells

**Figure 1:**
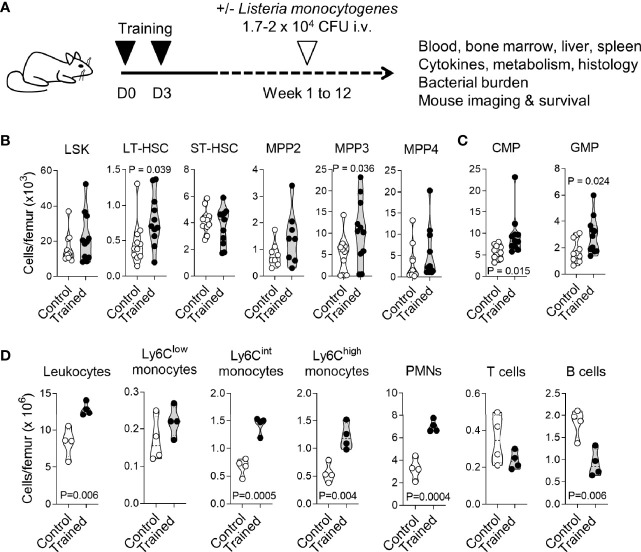
Training for 9 weeks promotes myelopoiesis. **(A)** Experimental model to study trained immunity. **(B, C)** Bone marrow cells were collected from control mice (*n* = 11) and mice trained 9 weeks (*n* = 4) and 12 weeks (*n* = 7) before analysis. Cell populations were analyzed by flow cytometry for hematopoietic progenitors (LSK), long-term hematopoietic stem cells (LT-HSC), short-term HSC (ST-HSC), multipotent progenitors (MPP) 2, MPP3 and MPP4 **(B)**, common myeloid progenitors (CMP) and granulocyte-monocyte progenitors (GMP) **(C)**. **(D)** CD45^+^ leukocytes, Ly6C^low^, Ly6C^lint^ and Ly6C^high^ monocytes (CD11b^+^Ly6G^-^), PMNs (Ly6G^+^), T cells (CD3^+^) and B cells (B220^+^) in the bone marrow from control mice (*n* = 4) and mice trained for 9 weeks (*n* = 4). Each dot of the violin plots represents data from one individual mouse, and the dashed horizontal lines the quartiles. *P* values are given in the graphs.

Experiments were performed with 8 to 10-week-old C57BL/6J female mice (Charles River Laboratories, Saint-Germain-sur-l’Arbresle, France). Mice were acclimated one week at least before usage. Mice were housed under specific pathogen-free and mouse hepatitis and mouse norovirus free conditions of the animal facility of the Centre des Laboratoires d’Epalinges (Switzerland, license VD-H04). Housing conditions were 21 ± 2°C, 55 ± 10% humidity, and 14-hours (h) light/10-h dark cycles. Food (SAFE or KILIBA NAGAF) and water (local filtered and autoclaved water or Innovive Aquavive^®^) were given ad libitum. Mice were injected intra-peritoneally (i.p.) twice at 4 days apart with 1 mg zymosan (Sigma-Aldrich, St-louis, MO) to induce training (**Figure 1**). Bone marrow cells were collected from femurs and tibias, while blood was collected from the submandibular vein in EDTA-tubes (Sarstedt, Nümbrech, Germany) (26, 27). Polymorphonuclear neutrophils (PMNs) and monocytes were isolated from the bone marrow of femurs and tibias using the mouse Neutrophil Isolation Kit and Monocyte Isolation Kit (BM) (Miltenyi Biotec, Bergisch Gladbach, Germany), respectively. The purity of the preparations, assessed by flow cytometry, was 85-90% for PMNs and 70-80% for monocytes.

### Reagents

Details about reagents, kits and antibodies used for flow cytometry analyses are reported in **Supplementary Information**. Zymosan A from *Saccharomyces cerevisiae* and phytohemagglutinin-L (PHA) from *Phaseolus vulgaris* were from Sigma-Aldrich, *Salmonella minnesota* ultra-pure lipopolysaccharide (LPS) from List Biologicals Laboratories (Campbell, CA), and CpG ODN 1826 (CpG) from Microsynth (Balgach, Switzerland). *Listeria monocytogenes* 10403S (*L. monocytogenes*) was grown in brain heart infusion broth (Thermo Fisher Scientific, Waltham, MA). *Candida albicans* 5102 (*C. albicans*) was cultured in yeast extract-peptone-dextrose (BD Biosciences, Franklin Lakes, NJ) (28). Microorganisms were washed with PBS and heat-inactivated for 2 h at 56°C [*C. albicans*; (28, 29)] or 70°C (*L. monocytogenes*, unpublished protocol).

### *In Vivo* Model of Listeriosis

Listeriosis was induced by challenging mice intravenously (i.v.) with 1-2 x 10^4^ CFU *L. monocytogenes*. The median lethal dose (LD_50_) of the *Listeria* strain in C57BL/6J female mice is around 0.1-0.5 x 10^4^ CFU. Blood was collected 2 days post-infection to quantify bacteria and cytokines and analyze cell populations. In some experiments, mice were sacrificed to collect bone marrow, liver and spleen. Body weight loss, severity score and survival were registered at least twice daily by 2-3 operators. The severity score was graded 0-5 based on mobility, posture, appearance and weight loss. The criteria were approved by the Service des Affaires Vétérinaires, DGAV, and are available upon request. Mice were sacrificed when they met a severity score of 4, while a dead mouse was assigned a score of 5 (30).

### Flow Cytometry

Bone marrow cells were incubated 5 minutes (min) on ice with 3 ml ice-cold red blood cell (RBC) lysis buffer (0.65 M ammonium chloride, 10 mM sodium bicarbonate, 0.1 mM EDTA, pH 7.4). Cells were washed with cell staining medium (CSM: PBS containing 0.5% BSA (Sigma-Aldrich)) and enumerated (29). Five times 10^5^-10^6^ cells were incubated 30 min in the dark at 4°C with 50 μl live/dead dye (Fixable Viability Dye eFluor™ 450 or LIVE/DEAD™ Fixable Violet, Thermo Fisher Scientific), then with 50 µl of 2.4G2 monoclonal antibody (cell culture supernatant of confluent cells, ATCC^®^ HB-197™, ATCC, Manassas, VA) and finally with 50 µl of antibodies diluted in CSM to identify bone marrow progenitors or leukocytes (23). Cells were washed, incubated 5 min with 5% paraformaldehyde (PFA, Thermo Fisher Scientific), washed and re-suspended in 1 ml CSM. For whole blood staining, 20 µl of whole blood were incubated 30 min in the dark at room temperature with 50 µl of antibodies diluted in CSM. Reaction mixtures were diluted with 500 µl of RBC lysis buffer and incubated for 10 min. Cells were washed, incubated 5 min with 5% PFA, washed and re-suspended in 1 ml CSM. Throughout procedures, samples were centrifuged for 5 min at 400 x g and 4°C, and washing steps were performed with 1 ml CSM. Spleens were dissociated in gentleMACS™ C Tubes using a gentleMACS Octo Dissociator (Miltenyi Biotec). Five x 10^5^ or 10^6^ splenocytes were treated as bone marrow cells for flow cytometry analyses.

Eight hundred microliters of cell suspensions (*i.e.* 4 x 10^6^ bone marrow cells and 0.4 x 10^6^ splenocytes) were acquired using an Attune NxT Flow Cytometer (Thermo Fisher Scientific) linked to an autosampler. Flow cytometry data were analyzed using FlowJo v.10 software (FlowJo LLC, Ashland, OR). Gating strategies were described previously (23, 30, 31). The antibody mixture to stain BM progenitor cells contained a lineage cocktail (anti-B220, CD3, CD11b, CD19, Ly-76 and Ly6C/G antibodies), and anti-CD16/32, CD34, CD117/c-kit and Ly6A/E/Sca-1 antibodies. The antibody mixture to stain BM and blood leukocytes contained anti-CD3, CD11b, CD19, CD45, Ly6C and Ly6G antibodies (**Supplementary Information**).

### *Ex Vivo* Blood Stimulation Assay

Twenty microliters of blood in 96-well plates were incubated with 80 μl of stimulus diluted in RPMI (Thermo Fisher Scientific). After 6 h or 24 h, reactions were mixed by gentle up and down pipetting and centrifuged for 5 min at 400 x g and 4°C. Supernatants were collected to quantify cytokines. PMNs and monocytes (10^5^ cells in 200 μl) in 96-well plates were incubated for 24 h with 100 ng/ml LPS or 10^8^ heat-killed *L. monocytogenes*. Following centrifugation, supernatants were collected to quantify cytokines.

### Cytokine and Myeloperoxidase Measurements

IL-6, TNF, IL-12p40 cytokines and MPO concentrations were determined with DuoSet ELISA kits (R&D systems, Minneapolis, MN) and IL-1β cytokine using ELISA Ready-SET-Go (eBioscience, San Diego, CA). The plates were read on a VersaMax Microplate Reader (Molecular Devices, San José, CA). A 14 Mouse Custom ProcartaPlex (Thermo Fisher Scientific) was used to determine the concentrations of G-CSF, IFNγ, IL-1α, IL-1β, IL-6, IL-10, IL-12p40, IL-18, TNF, CCL2, CCL3, CXCL2, CXCL5 and CXCL10 using a Bioplex 200 system (Bio-Rad, Hercules, CA) (29).

### Bacterial Growth Assay and Bacteria Quantification

Twenty microliters of blood in 96-well plates were incubated for 2 h with 6 x 10^4^ CFU *L. monocytogenes* in 130 μl RPMI without additives. Plates were shacked for 1 min at 400 rpm using a microplate shaker (Edmund Buhler, Bodelshausen, Germany) and serial dilutions of blood were plated on Columbia III Agar with 5% Sheep blood medium (BD Biosciences). Plates were incubated for 24 h at 37°C, and colonies were enumerated. Given that *L. monocytogenes* is an intracellular Gram-positive bacterium, we verified that similar CFUs were obtained using native blood and lysed blood samples (native/lysed x 100: 106.9 ± 15.1%, *n* = 8, *P* > 0.1). One hundred thousand PMNs or monocytes in 1.5 ml tubes were incubated for 2 h with 10^3^ CFU *L. monocytogenes* in 200 μl RPMI containing 2% murine plasma. Tubes were centrifuged 10 min at 4,100 × g. Pellets were resuspended in 200 μl sterile water and incubated for 10 min to lyse cells. Spleens were homogenized with an UltraTurrax homogenizer (IKA-Werke, Staufen, Germany). CFUs were determined as described above.

### RNA Analyses

Total RNA was isolated, reverse transcribed and used in real-time PCR analyses performed using a QuantStudio™ 12K Flex system (Life Technologies, Carlsbad, CA) (29). Reactions, tested in triplicates, consisted of 2 µl cDNA, 2 µl H_2_O, 1 µl 0.5 µM primers and 5 µl Fast SYBR^®^ Green Master Mix (Life Technologies). Gene expression was analyzed using the threshold cycle (C_T_) method 2^-ΔΔCt^. Data were normalized to *Actin* expression and were reported to the expression in control PMNs set at 1. Primers are listed in **Supplementary Information**.

### Migration Assay

Cell migration was assessed using Corning Costar Transwell^®^ cell culture inserts with 5 μM pore size (Corning B.V. Life Sciences, Amsterdam, NL) as described previously (32). Briefly, 3 x 10^5^ PMNs were resuspended in HBSS medium (Invitrogen) containing 0.1% BSA (Sigma-Aldrich, Buchs, Switzerland) and transferred to the transwell inserts. The lower chamber of the transwell device contained medium with or without 25 nM recombinant murine CXCL2/MIP2 (Preprotech, Rocky Hill, NJ). The number of cells migrating in the lower chamber was assessed after 1 h using an Attune NxT Flow Cytometer (Thermo Fisher Scientific).

### Metabolic Activity

Metabolic parameters were determined using a Seahorse XFe96 Analyzer (Agilent, Santa Clara, CA) (33). PMNs and monocytes, isolated from the bone marrow, were seeded at 1.5 x 10^5^ cells per well in Seahorse XFe96 plates (Agilent) pre-coated with 22.4 µg/ml CellTak (Corning, Corning, NY) diluted in 0.1 M sodium bicarbonate pH 8.0 (Sigma-Aldrich). Cells were plated in assay medium (Seahorse XF DMEM pH 7.4 supplemented with 2 mM glutamine, 1 mM pyruvate and 10 or 25 mM glucose (Agilent) for glycolytic activity or mitochondrial respiration, respectively). PMNs and monocytes were incubated for 30 min with or without 10^8^ heat-killed *L monocytogenes* in assay medium at 37°C without CO_2_. Glycolytic activity, depicted by means of proton efflux rate (glycoPER), was assessed following Seahorse Cell Glycolytic Rate protocol (user guide kit 103344-100, Agilent), which consists of the sequential addition of 0.5/0.5 µM rotenone + antimycin A (Rot/AA) and 50 mM 2-deoxyglucose (2-DG, Sigma-Aldrich). Mitochondrial respiration, depicted by means of oxygen consumption rate (OCR), was assessed following Seahorse Mito Stress protocol (user guide kit 103015-100, Agilent), which consists of subsequent injections of 1 µM oligomycin (oligo), 2 µM carbonyl cyanide 4-(trifluoromethoxy) phenylhydrazone (FCCP) and 0.5/0.5 µM Rot/AA (Sigma-Aldrich). Results were analyzed using Wave Desktop software (Agilent). Cells were lysed with a mixture of RIPA Buffer IV (Bio Basic, Markham, Canada) and a complete protease inhibitor cocktail tablet (Roche Life Science, Basel, Switzerland) in PBS. Data were normalized to protein content quantified using the Pierce BCA Protein Assay Kit (Thermo Fisher Scientific). Data are represented as means ± SEM from 4 mice analyzed in quadruplicate.

### Quantification of Inflammation *In Vivo* by Bioluminescence Imaging

Mice were injected i.p. with 200 mg/kg luminol (Carbosynth, Staad, Switzerland). After 10 min, mice were anesthetized using isoflurane in a veterinary induction chamber for rodents (PS-0346, Rothacher Medical GmbH, Heitenried, Germany) and transferred to an In-Vivo XtremeTM II SPF animal chamber with high-efficiency particulate air (HEPA) filters (BRUKER, Billerica, MA). Image acquisition was performed using an In-Vivo Xtreme II system (BRUKER) with luminescence and X-Ray modalities. The capture values for luminescence modality were 10 min exposure time, binning 8x8 pixels, fSTOP 1.1 and field of view (FOV) and for X-Ray modality parameters were 5 sec exposure time, binning 1x1 pixels, fSTOP 4 and FOV 19 cm. Images were analyzed using the Molecular Imaging software v.7.5.3 from BRUKER. Regions of interest (ROI) were set on the upper peritoneal cavity and the net bioluminescent signals were expressed in units of photons per second (P/s).

### Histology

Liver was fixed overnight in 4% PFA, washed with PBS and embedded into paraffin. Transversal liver sections 5 µm thick were stained with hematoxylin and eosin (H&E) at the Mouse Pathology Facility of the University of Lausanne (Epalinges, Switzerland). Images were acquired using an EVOS M7000 Imaging System (Thermo Fisher Scientific) with Plan Apochromat 2x and 10x objectives using bright field contrast. Representative images of sections are shown. Liver sections were reconstructed to quantify the number of foci. The size and number of foci were determined using EVOS™ Analysis software v.1.4.

### Statistical Analyses

Data were analyzed using PRISM v.8.3.0 software (GraphPad Software, La Jolla, CA, USA) and R software v.3.6.0 (R Foundation for Statistical Computing, Vienna, Austria). Groups were compared by two-tailed unpaired t tests or by ANOVA followed by Dunnett’s multiple comparisons test. Mouse survival was analyzed using the Kaplan-Meier method. *P* values < 0.05 were considered to be statistically significant. *, *P* < 0.05; **, *P* ≤ 0.01; ***, *P* ≤ 0.001; ****, *P* ≤ 0.0001 unless *P* values are mentioned in figures.

## Results

### Training for 9 Weeks Sustains Myelopoiesis

We have shown that training with zymosan, a fungal cell wall preparation rich in β-glucan, protected mice from lethal sepsis when infection was initiated 1 week after the induction of training (23). To determine whether trained immunity persists for a longer period, we compared control mice with mice trained 9-12 weeks before analysis (long term training), unless specified otherwise (**Figure 1A**). We analyzed immune and progenitor cells in bone marrow and peripheral blood compartments. In the bone marrow, the number of LSK (Lin^-^Sca1^+^cKit^+^) hematopoietic progenitors, short-term (ST) HSCs (CD48^-^CD150^-^ LSKs) (**Figure 1B**), myeloid-biased multipotent progenitors (MPPs) MPP2 (Flt3^-^CD48^+^CD150^+^ LSKs) and lymphoid-biased MPP4 (Flt3^+^CD48^+^CD150^-^ LSKs) were similar in control mice and in trained mice. On the contrary, the number of long-term (LT) HSCs (CD48^-^CD150^+^ LSKs) and myeloid-biased MPP3 (Flt3^-^CD48^+^CD150^-^ LSKs) was increased 1.7 and 2.2-fold in trained mice (*P* = 0.039 and 0.036, respectively) (**Figure 1B**). In line with these results, common myeloid progenitors (CMPs) and granulocyte-monocyte progenitors (GMPs) were increased 1.7-fold in the bone marrow of trained mice (*P* = 0.015 and 0.024) (**Figure 1C**). We then compared bone marrow leukocyte subpopulations (**Figure 1D**). Following increased myelopoiesis, the bone marrow from mice trained for 9 weeks contained 1.6-fold more CD45^+^ leukocytes (*P* = 0.006) (**Figure 1D**). This increase resulted from an increase of PMNs and to a lesser extent of Ly6C^high^ inflammatory/classical monocytes and Ly6C^int^ monocytes (3.1, 2.1, 2.2-fold-increase, *P* < 0.005). The counts of Ly6C^low^ nonclassical/patrolling monocytes and T cells were not altered, while the counts of B cells were reduced 2-fold (*P* = 0.006). The frequency of LT/ST-HSCs and MPP subpopulations within LSK cells and of leukocyte subpopulations within total leukocytes are given in **Figure S1**.

**Figure 2:**
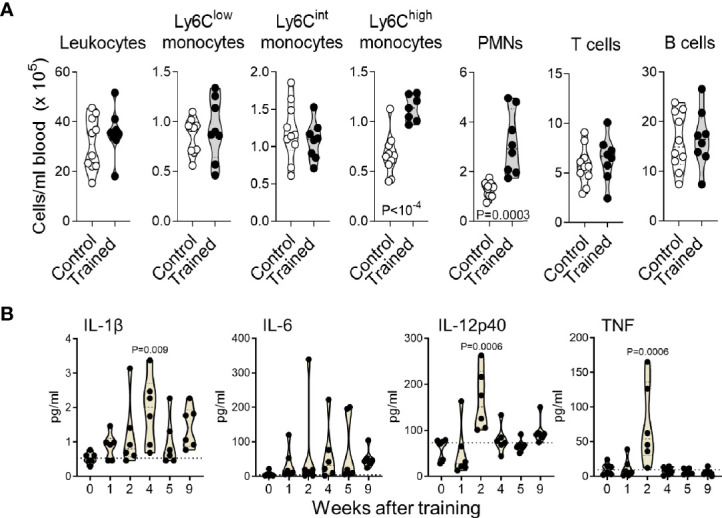
Training for 9 weeks increases blood inflammatory monocytes and PMNs. **(A)** Blood counts of CD45^+^ leukocytes, Ly6C^low^, Ly6C^lint^ and Ly6C^high^ monocytes (CD11b^+^Ly6G^-^), PMNs (Ly6G^+^), T cells (CD3^+^) and B cells (B220^+^) in control mice (*n* = 10) and mice trained for 9 weeks (*n* = 8). Data are from 2 independent experiments. **(B)** Blood concentrations of IL-1β, IL-6, IL-12p40 and TNF in control mice and mice trained 1-9 weeks before analysis (*n* = 6 mice/group). Each dot represents a mouse. Lines represent mean and SD. The dotted horizontal lines in B represent median and limit of detection. *P* values reaching statistical significance are given in the graphs.

The blood from mice trained for 9 weeks contained 1.7 and 2.4-fold more Ly6C^high^ monocytes and PMNs (*P* < 10^-4^ and *P* = 0.0003), while the counts of Ly6C^low/int^ monocytes, T cells and B cells were not affected (**Figure 2A**). IL-1β is an important regulator of hematopoiesis (34). It has been involved in the induction of trained immunity induced with BCG, β-glucan and Western diet and to promote the proliferation of HSCs (21, 35, 36). Thus, we quantified IL-1β in the blood of mice trained for 0, 1, 2, 4, 5 and 9 weeks (**Figure 2B**). Interestingly, IL-1β concentrations were 1.6-3.6 higher in trained mice than in control mice. The highest concentrations were measured 4 weeks after training (*P* = 0.009 versus control). We also measured IL-6, IL-12p40 and TNF to consider systemic inflammation induced by trained immunity. IL-6 was slightly increased in some mice, but results were not statically significantly different from control mice. IL-12p40 and TNF were at highest (but low) levels 2 weeks after training (*P* = 0.0006 versus control) and were back to control levels in mice trained 4 weeks onwards (**Figure 2B**).

To test whether PMNs and monocytes could be responsible for the elevated levels of cytokines, particularly IL-1β, we measured cytokine production by bone marrow PMNs and monocytes (**Figure S2**). Unstimulated PMNs and monocytes did not release detectable levels of IL-1β, IL-6 and TNF. When compared to cells from control mice, PMNs and monocytes from trained mice released higher levels of IL-6 in response to LPS stimulation (positive control of training) and similar levels of TNF in response to LPS and *L. monocytogenes*. IL-1β release was weakly induced by LPS only, and to similar levels in control and trained cells. Thus, bone marrow PMNs and monocytes might not be responsible for the elevated IL-1β levels. Overall, these data indicate that training increased myelopoiesis and Ly6C^high^ monocytes and PMNs counts in peripheral blood for up to 9 weeks.

### Training for 9 Weeks Sustains the Antimicrobial Response of Whole Blood

**Figure 3:**
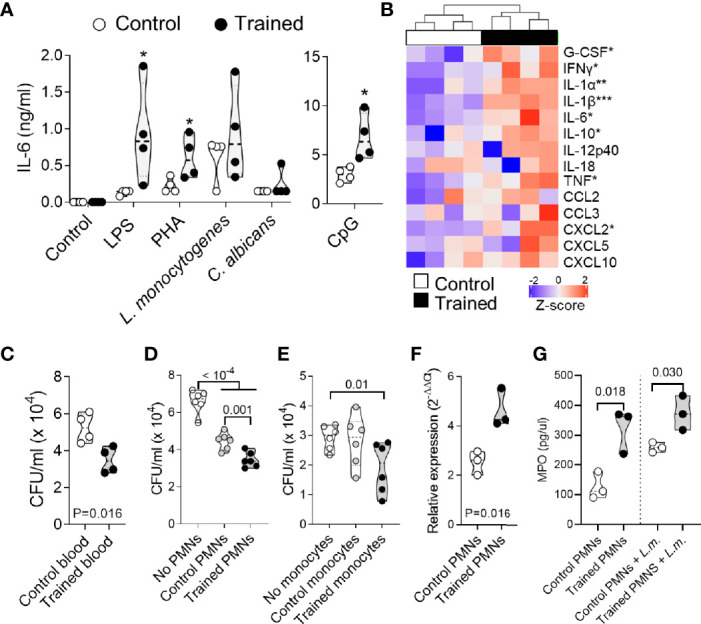
Training for 9 weeks promotes whole blood antimicrobial responses. Blood was collected from control mice and mice trained 9 weeks before collection (*n* = 4-5 mice per group). **(A, B)** Blood was incubated for 6 h with 10 ng/ml LPS, 1 μg/ml PHA, 10 μM CpG and 10^8^ heat-killed *L. monocytogenes* and *C. albicans*. The concentration of IL-6 in blood supernatants was quantified by ELISA. Each dot of the violin plots represents data from one individual mouse, and the dashed horizontal lines the median. *P < 0.05 **(A)**. The concentrations of G-CSF, IFNγ, IL-1α, IL-1β, IL-6, IL-10, IL-12p40, IL-18, TNF, CCL2, CCL3, CXCL2, CXCL5 and CXCL10 in blood supernatants were quantified by multiplex immunoassay (Luminex). Heatmap and hierarchical clustering of cytokine levels was performed using Z-score normalization **(B)**. **(C–E)** Blood **(C)**, PMNs **(D)** and monocytes **(E)** were incubated for 2 h with 6 x 10^4^, 6 x 10^3^ and 1.8 x 10^3^ CFU *L. monocytogenes*, respectively. Serial dilutions of blood or samples of lysed cells were plated on blood agar plates. After and incubation for 24 h, CFUs were enumerated **(F, G)** PMNs were culture 1 h with or without 10^8^ heat-killed *L. monocytogenes*. Myeloperoxidase (*Mpo*) relative mRNA levels were measured by RT-PCR and normalized to *actin* housekeeping gene expression **(F)**. MPO concentrations in cell culture supernatants were quantified by ELISA **(G)**. Each dot in the violin plots represents data from one individual mouse and the dashed horizontal lines the quartiles. *P < 0.05; **P < 0.01; ***P < 0.001 *vs*. control **(A, B)**.

Leukocytes form a major barrier against systemic infection. Hence, we tested the reactivity of whole blood collected from control mice and trained mice against a range of stimuli including LPS, PHA, CpG, and heat killed *L. monocytogenes* and *C. albicans* (**Figure 3A**). The blood of mice trained 9 weeks before collection produced more IL-6 upon exposure to LPS, PHA and CpG (*P* < 0.05) than the blood of control mice. IL-6 levels were higher also in response to heat-killed *L. monocytogenes* and *C. albicans*, though differences did not reach statistical significance. To extend our analyses, we performed a multiplex immunoassay (Luminex) to quantify 14 cytokines (G-CSF, IFNγ, IL-1α, IL-1β, IL-6, IL-10, IL-12p40, IL-18, TNF, CCL2, CCL3, CXCL2, CXCL5 and CXCL10) expressed by whole blood exposed to LPS (**Figure 3B**). Globally, the blood from trained mice produced more cytokines. Differences were statistically significant for G-CSF, IFNγ, IL-1α, IL-1β, IL-6, IL-10, TNF and CXCL2.

To mimic infectious conditions, we measured the capacity of whole blood to limit the growth of live *L. monocytogenes*. Blood was incubated for 2 h with *L. monocytogenes*, and the number of bacteria was quantified (**Figure 3C**). The blood from trained mice contained more efficiently the growth of *L. monocytogenes*, as shown by 1.5-fold lower bacterial counts in the blood from trained mice than from control mice (*P* = 0.016). We then analyzed the killing capacity of PMNs and monocytes which are the main phagocytic cells present in blood (**Figures 3D, E**). PMNs and monocytes from trained mice contained more efficiently the growth of *L. monocytogenes*. CFUs were 1.4 fold lower using PMNs from trained mice when compared to control PMNs (*P* = 0.001) (**Figure 3D**), and 1.5-fold lower using monocytes from trained mice when compared to conditions without monocytes (*P* = 0.01) (**Figure 3E**). In line with an increased killing capacity of PMNs, PMNs from trained mice expressed higher levels of myeloperoxidase (MPO) mRNA (*P* = 0.016) (**Figure 3F**) and secreted higher levels of MPO at baseline and upon exposure to *L. monocytogenes* (**Figure 3G**) when compared to PMNs from control mice (*P* = 0.018 and 0.030, respectively).

### Impact of Training on Leukocyte Counts in Blood and Spleen

**Figure 4:**
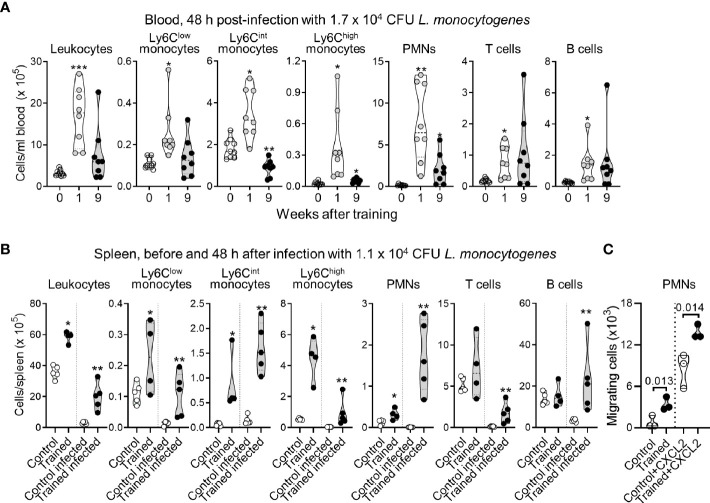
Impact of training on leukocyte counts in blood and spleen during listeriosis. **(A)** Control mice (*n* = 9) and mice trained 1 week and 9 weeks before infection (*n* = 8 in each group) were challenged i.v. with 1.7 x 10^5^ CFU *L. monocytogenes*. Blood was collected 2 days after infection to quantify CD45^+^ leukocytes, Ly6C^low^, Ly6C^lint^ and Ly6C^high^ monocytes (CD11b^+^Ly6G^-^), PMNs (Ly6G^+^), T cells (CD3^+^) and B cells (B220^+^). Each dot in the violin plots represents data from one individual mouse, and the dashed horizontal lines the quartiles. **(B)** Control mice and mice trained 1 week before infection were challenged i.v. with 1.1 x 10^5^ CFU *L. monocytogenes*. Spleens were collected before infection (*n* = 4-5/group) and 2 days after infection (*n* = 5 mice/group) to quantify CD45^+^ leukocytes, Ly6C^low^, Ly6C^lint^ and Ly6C^high^ monocytes, PMNs, T cells and B cells. Each dot of the violin plots represents data from one individual mouse, and the dashed horizontal lines the quartiles. **(C)** PMNs were isolated from control mice and mice trained 9 weeks before analysis. Spontaneous and CXCL2-dependent migration of PMNs was measured in transwell assay. CXCL2 was used at 25 nM. Migrating cells were enumerated after 1 h. Each dot in the violin plots represents data from one individual mouse. *P < 0.05; **P < 0.01; ***P < 0.001.

Our observations suggested that mice trained for 9 weeks should be protected from infection. To verify this assumption, we used a preclinical model of sepsis to compare the behavior of control mice to that of mice trained 9 weeks before analysis. In some experiments we included mice trained 1 week before analysis, used as a reference (23). Mice were infected i.v. with 1-2 x 10^4^ CFU *L. monocytogenes*, corresponding to around 2-20 times control mice LD_50_. Two days post-infection, listeriosis induced a profound depletion of peripheral blood leukocytes (around 10-fold decrease, compare cell counts in **Figures 2** and **4**). Training 1 week prior to infection sustained leukocyte counts (*P* < 0.01), including those of Ly6C^low^ monocytes, Ly6C^int^ monocytes, Ly6C^high^ monocytes, PMNs, T cells and B cells (*P* < 0.01-10^-4^). Training 9 weeks prior to infection also sustained leukocyte counts, albeit less efficiently than training for 1 week. Results were statistically significant for Ly6C^high^ monocytes (*P* < 0.01), PMNs (*P* < 0.001) and T cells (*P* < 0.05). B cell counts were heterogeneous but higher (around 6-fold) in mice trained for 9 weeks than in control mice (*P* = 0.09), while Ly6C^int^ monocytes were decreased around 2-fold (*P* < 10^-3^) (**Figure 4A**).

We then compared the numbers of leukocytes in the spleen of control and trained mice to assess whether variation in blood cell numbers reflected cell destruction or cell migration in an organ that *L*. *monocytogenes* infects (**Figure 4B**). Indeed, bacteria colonized the spleen and were around 500-fold more abundant in control mice than in trained mice (1.08 ± 0.66 x 10^6^ versus 0.002 ± 0.002 x 10^6^ CFU/gr spleen, *n* = 4/5, *P* = 0.008). Before infection, when compared to control conditions, training increased leukocytes, Ly6C^low^, Ly6C^int^ and Ly6C^high^ monocytes, and PMNs (*P* < 0.05) in the spleen. Two days post-infection, leukocytes including Ly6C^low^ and Ly6C^high^ monocytes and T cells were reduced 10-fold in control mice and 5-fold in trained mice. B cells and PMNs were reduced 3.8-fold in control mice, while Ly6C^int^ slightly increased in control and trained mice. Interestingly, PMNs were reduced 15.8-fold in control mice (*P* = 0.002), while they were increased 5-fold in trained mice (*P* = 0.003) (**Figure 4B**), suggesting that the induction of training supports the migration of PMNs in peripheral organ.

To verify that training may promote the migration of PMNs, PMNs from control and trained mice were subjected to transwell migration assays performed in the presence or in the absence of the inflammatory chemokine CXCL2/MIP2α shown to be strongly increased by whole blood from trained mice exposed to LPS (**Figure 3B**). The number of migrating cells was assessed after 1 h of incubation. As shown in **Figure 4C**, CXCL2 increased the migration of PMNs by a factor of 4 to 10 in control and trained mice. Overall, spontaneous and CXCL2-induced chemotaxis of PMNs from trained mice were 4.2- and 1.6-fold higher than that of control PMNs (*P* = 0.013 and 0.014, respectively).

### Impact of Training on the Metabolism of PMNs and Monocytes

**Figure 5:**
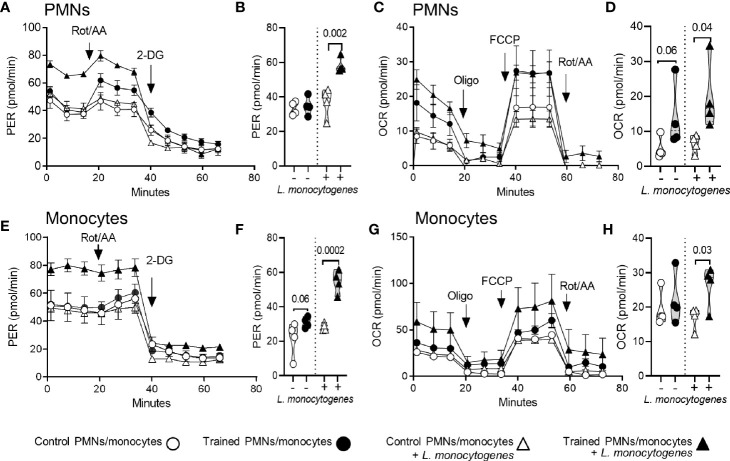
Training for 9 weeks increases the metabolic activity of PMNs and monocytes. PMNs and monocytes were isolated from the bone marrow of control mice and mice trained 9 weeks before analysis (*n* = 4 mice per group). One hundred fifty thousand PMNs **(A–D)** and monocytes **(E–H)** were incubated for 30 min with or without 10^8^ heat-killed *L monocytogenes* before measuring glycolytic activity **(A, E)** and mitochondrial respiration **(C, G)**. Glycolytic activity was determined using Glycolytic Rate kit, with consecutive injections of 0.5/0.5 μM Rot/AA (rotenone/antimycin A) and 50 mM 2-DG (2-deoxyglucose). Glycolytic activity is depicted by means of proton efflux rate (PER) adjusted to protein concentration per condition. Mitochondrial respiration was analyzed using Mito Stress kit, with consecutive injections of 1 µM oligo (oligomycin), 2 µM FCCP and 0.5/0.5 μM Rot/AA. Mitochondrial respiration is depicted by means of oxygen consumption rate (OCR) adjusted to protein concentration per condition and represented as OCR. **(B, F)** Basal glycolysis (*i.e.* last PER measurement before the first injection) expressed by the PER in pmol/min. **(D, H)** Basal respiration (*i.e.* energetic demand of the cell under baseline conditions) expressed by the OCR in pmol/min. Data are means ± SEM from 4 mice analyzed in quadruplicate. Each dot in the violin plots represents data from one individual mouse.

The induction of trained immunity has been associated with metabolic rewiring in myeloid cells, favoring aerobic glycolysis over oxidative phosphorylation (OXPHOS) (37–41). Therefore, we compared the metabolic parameters of PMNs and monocytes isolated from the bone marrow of control mice and mice trained for 9 weeks (**Figure 5**). PMNs and monocytes were incubated with or without *L. monocytogenes* before measuring the glycolytic rate (**Figures 5A, B, E, F**) and mitochondrial respiration (**Figures 5C, D, G, H**) of the cells using the Seahorse technology. *L. monocytogenes* strongly increased the glycolytic rate of trained PMNs and monocytes, while it more subtly increased the respiration rates of the cells and the metabolic parameters of control PMNs and monocytes. Interestingly, PMNs from trained mice showed higher glycolytic rate (**Figures 5A, B**) and mitochondrial respiration (**Figures 5C, D**) upon exposure to *L. monocytogenes* (1.6-fold and 3.1-fold increase *vs* control PMNs, *P* = 0.002 and 0.04, respectively). In a similar way, monocytes from trained mice showed higher glycolytic rate (**Figures 5E, F**) and oxidative metabolism (**Figures 5G, H**) upon exposure to *L. monocytogenes* (1.9-fold and 1.5-fold increase *vs* control PMNs, *P* = 0.0002 and 0.03, respectively). These results support the assumption that training promotes glycolytic metabolism and, less markedly, OXPHOS of phagocytic cells to sustain antimicrobial activity.

### Training for 9 Weeks Protects From Listeriosis

**Figure 6:**
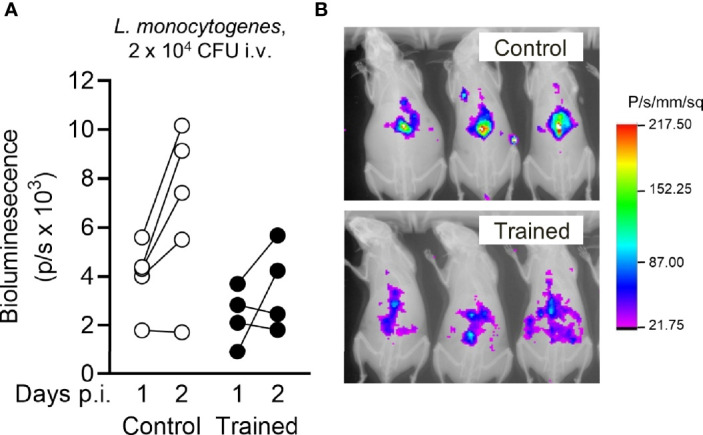
Training for 9 weeks reduces *Listeria*-induced inflammation measured by bioluminescence imaging. Control mice and mice trained 9 weeks before infection (*n* = 4 mice per group) were challenged i.v. with 1.7 x 10^4^ CFU *L. monocytogenes*. One day and 2 days post infection, inflammatory levels were measured by luminol-generated bioluminescence using an imaging system. **(A)** Quantification of bioluminescent signals. Each dot represents a mouse. Lines connect results obtained from one animal analyzed 1 and 2 days post infection (p.i.). **(B)** Representative images of control and trained mice analyzed 2 days p.i. displayed as an overlay of luminescent and X Ray modalities. Control *versus* trained at day 1 and day 2: *P* = 0.052 and 0.062, respectively.

To visualize mouse response to infection with *L. monocytogenes*, we performed *in vivo* bioluminescence imaging. One and 2 days post-infection, mice were injected with luminol that reacts with superoxide generated in phagosomes, reflecting the myeloperoxidase activity of tissue-infiltrating neutrophils. As shown in **Figure 6**, luminol-mediated bioluminescent signals were detected in the abdomen of mice, which was in agreement with the rapid colonization of liver upon systemic infection with *L. monocytogenes*. The signals were globally lower in mice trained 9 weeks before infection than in control mice (*P* = 0.05 and 0.06 at days 1 and 2 post-infection). It likely reflected lower bacteria spreading and subsequent antimicrobial inflammatory response in the organs of trained mice.

**Figure 7:**
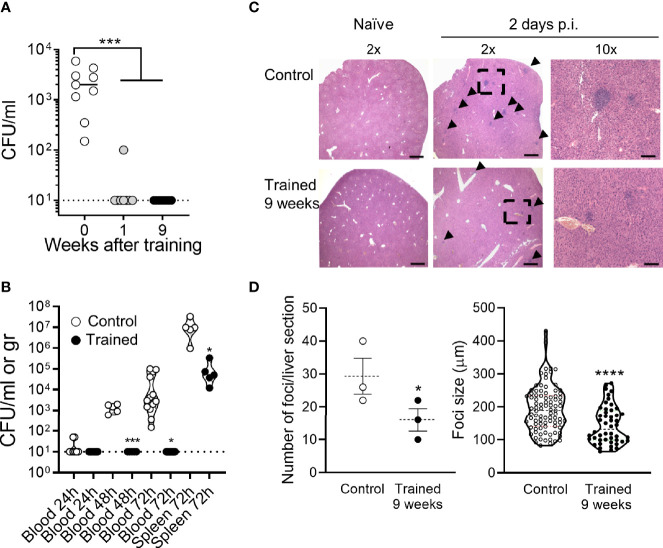
Training for 9 weeks prevents *L. monocytogenes* burden and dissemination. Control mice and mice trained 1 week or 9 weeks before infection were challenged i.v. with 1.5-2 x 10^4^ CFU *L. monocytogenes*. **(A)** Blood was collected 2 days p.i. to quantify bacteria. Each dot represents a mouse (*n* = 9, 8 and 8, for mice trained for 0, 1, or 9 weeks, respectively). The plain and dotted horizontal lines represent median and limit of detection, respectively. **(B)** Blood and spleen were collected 1-3 days p.i. to quantify bacteria. Each dot represents a mouse (n = 4-13). The plain and dotted horizontal lines represent median and limit of detection, respectively. **(C, D)** Livers (*n* = 3 control mice and 3 mice trained 9 weeks) were collected 2 days p.i. and liver sections stained with hematoxylin and eosin. **(C)** Representative images of liver sections imaged at 2x and 10x magnification using an EVOS M7000 microscope. Scale bar represents 750 µm or 150 µm for 2x and 10x magnification images, respectively. Arrows point to foci and the doted square to the area zoomed 10x. **(D)** Number of foci per liver section and size of foci. *P < 0.05; ***P < 0.001; ****P < 0.0001 *vs.* control.

**Figure 8:**
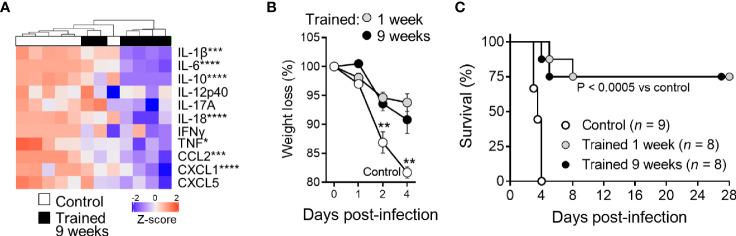
Training for 9 weeks increases mice survival from lethal listeriosis. Control mice and mice trained 1 week and 9 weeks before infection were challenged i.v. with 1.7 x 10^4^ CFU *L. monocytogenes*. **(A)** Blood was collected 2 days after infection to quantify cytokines (IL-1β, IL-6, IL-10, IL-12p40, IL-17A, IL-18, IFNγ, TNF, CCL2, CXCL1, and CXCL5). Heatmap and hierarchical clustering of cytokine levels was performed using Z-score normalization. **(B, C)** Weight loss (expressed in percentage of initial weight) and survival of mice. Data are means ± SD **(B)**. *P < 0.05; **P < 0.01; ***P < 0.001; ****P < 0.0001 *vs.* control.

Two days post infection all control mice were strongly bacteremic, while mice trained 1 week and 9 weeks before infection were all but one sterile (*P <*10^-4^) (**Figure 7A**). The efficient control of bacterial dissemination burden was further attested by the fact that the blood from mice trained 9 weeks before infection did not contain detectable CFUs at days 1-3 post-infection (**Figure 7B**). Furthermore, the spleen of trained mice contained 2 Log less bacteria at day 3 (**Figure 7B**). To confirm these observations, we performed histology on the liver (**Figures 7C, D**). The number and the size of inflammatory foci were 1.8- and 1.3-fold lower in mice trained 9 weeks before infection than in control mice, respectively (*P* = 0.05 and *P <*10^-4^). Suggesting low systemic response, the blood of mice trained 9 weeks contained lower levels of a broad range of cytokines (IL-1β, IL-6, IL-10, IL-12p40, IL-17A, IL-18, IFNγ, TNF, CCL2 and CXCL5) 2 days after infection with *L. monocytogenes* (**Figure 8A**). Control mice lost more weight than mice trained 1 week or 9 weeks before infection (*P* < 0.01 *vs.* control) (**Figure 8B**). Accordingly, while all control mice died of infection within 4 days, 75% of mice trained for 1 or 9 weeks survived the infection (1 and 9 weeks of training *vs.* control: *P* < 10^-4^ and *P* = 0.0003, respectively) (**Figure 8C**).

## Discussion

Trained immunity protects from candidiasis and from chronic and acute bacterial infections (16, 23). Extending these observations, we report that, 9 weeks after the induction of trained immunity, mice were still fully protected in a model of lethal listeriosis. This survival advantage was associated with increased myelopoiesis and blood leukocytes especially PMNs, and metabolic rewiring in PMNs.

The blood from mice trained 9 weeks produced more cytokines upon stimulation with PAMPs and mitogen, and restricted more efficiently the growth of *L. monocytogenes* than blood from control mice. PMNs and monocytes from trained mice also displayed an increased killing activity. Along with this observation, monocytes/macrophages trained with β-glucan or BCG produced increased levels of cytokines when exposed to microbial compounds and displayed superior microbicidal responses (12, 16, 18, 21, 28, 42). The short lifetime of PMNs and monocytes raised the question of how innate immune memory persists over time in these cells. This was answered when β-glucan and BCG were shown to reprogram bone marrow HSCs generating trained myeloid cells (12, 13, 35).

Additional innate immune cells may acquire memory properties and participate to host defense responses. For example, memory skills have been attributed to microglia, dendritic cells, liver resident group 1 innate lymphoid cells (ILC1) and lung resident ILC2 obtained from mice exposed to LPS, *Cryptococcus neoformans*, cytomegalovirus, allergens and IL-33 (7, 10, 38, 43–47). Of great interest, the concept of training has been extended to non-immune cells. For instance, skin and respiratory epithelial progenitor stem cells exposed to inflammatory pressure as well as fibroblasts exposed to IFNβ display memory characteristics (14, 15, 48). Stem cells promote immune cell recruitment and repair mechanisms when they sense breaches in epithelial barriers. Moreover, stem cells respond to signals from recruited immune cells. This suggests bidirectional communication between stroma, stem cells and immune cells to optimize defense responses in trained conditions.

Nine weeks of training persistently stimulated myelopoiesis as demonstrated by increased bone marrow CMPs and GMPs, supporting a central role of HSCs in the establishment of a functional trained phenotype. The increase of myeloid progenitors paralleled the increase of Ly6C^high^ inflammatory monocytes and PMNs in peripheral blood, and the resistance of trained mice to listeriosis. Besides protecting from infections, PMNs are involved in pathophysiological processes. Interestingly, PMNs from mice trained with β-glucan promoted anti-tumor activity (49). Given that PMNs are abundant in the tumor microenvironment and that BCG vaccination enhanced the functions of blood PMNs in humans (50), trained immunity might be targeted for cancer treatment (51).

Upon recognition of DAMPs and PAMPs, cells undergo a metabolic shift to meet energy requirements for prompt responses. As archetypal examples, inflammatory macrophages and PMNs favor aerobic glycolysis over OXPHOS, while anti-inflammatory macrophages rely on fatty acid oxidation and the TCA cycle to generate ATP (52). The induction of trained immunity rewired the metabolism of bone marrow progenitors and monocytes, which was characterized by an increase of glycolysis and cholesterol metabolism (13, 37, 38). When compared to control cells, PMNs and monocytes from mice trained 9 weeks before analysis barely showed increased baseline metabolic parameters, while their glycolytic and respiration rates were strongly increased when exposed to *L. monocytogenes*. This suggests that long-term training sensitized phagocytes to increase their metabolism upon stimulation, which may help fighting bacterial infection. *Listeria* is an intracellular Gram-positive bacterium. When it infects cells, *Listeria* secretes a pore-forming toxin, listeriolysin O, which induces mitochondrial fragmentation to promote *Listeria* intracellular survival (53). It would be interesting to define whether training modifies mitochondrial content and protects mitochondria from the effects of listeriolysin O.

IL-1β circulated at low, but increased levels in the peripheral blood of mice up to 9 weeks after the induction of trained immunity. IL-1β promoted the proliferation of HSPCs and myelopoiesis as well as innate immune responses of monocytes (13, 34–36). Blocking IL-1 signaling through pharmacological or genetic approaches (using the IL-1 receptor antagonist anakinra or using *Il1r* or *Nlrp3* knockout mice) blunted the acquisition of a trained phenotype in mice fed with a Western diet or challenged with BCG, and reduced resistance to mycobacterial infection and listeriosis. Furthermore, functional and genetic studies in healthy volunteers suggested that IL-1β underlined the establishment of trained immunity induced by BCG and oxidized low-density lipoprotein (21, 23, 36, 54). Altogether, IL-1β may represent a hub regulating trained immunity induced by PAMPs and DAMPs. Considering that targeting IL-1 signaling has been proposed to represent an efficient way to modulate trained immunity pathways (55), it will be important to identify the primary source of IL-1β detected during the induction of training, and to determine how long subnormal levels of IL-1β persist in the circulation after training induction. Possibly, minutes levels of IL-1β may act locally to stimulate myelopoiesis or have an important biological impact.

Mice trained 9 weeks before infection cleared bacteria and survived upon high-dose infection by *L. monocytogenes*. Furthermore, blood, spleen and liver collected up to 2 months after the infectious challenge were sterile (data not shown), suggesting effective bacterial clearance in trained mice. The adoptive transfer in naive mice of bone marrow cells or long-term HSCs collected from mice 4 weeks after the induction of training increased the proportion of blood Gr1^+^CD11b^+^ myeloid cells and protected from pulmonary tuberculosis (12, 13). Alveolar macrophages showed marks of trained immunity 16 weeks following an intranasal challenge of adenovirus (7). Moreover, LPS-induced innate immune memory persisted 6 months in microglia (10). In healthy humans, BCG vaccination generated PBMCs, monocytes and neutrophils with increased enhanced effector functions 3 months after inoculation, and protected from experimental infection with the yellow fever vaccine given 4 weeks after BCG (8, 9, 21, 50). In a similar way, we report that in mice trained 9 weeks PMNs and monocytes had enhanced anti-microbial activities. The question arises whether PMNs or monocytes contribute (primarily) to protect trained mice from listeriosis. While we suspect that both cell types should play an important role, strategies based on cell depletion or blocking and adoptive cell transfer will help to settle the relative contribution of each cell type. Of note, other cell types such as dendritic cells, Kupffer cells, NK cells may play a role. Taken all together, these models support the assumption that trained immunity has persistent effects on immune responses in humans and mice.

Intriguingly, BCG vaccination promoted granulopoiesis and protected neonate mice from polymicrobial sepsis 3 days but not 13 days after vaccination. However, the protected effect of BCG vaccination vanished when infection was delayed by 10 days (56). This might indicate that, in this specific mouse model, BCG protected from polymicrobial sepsis in a narrow time window around birth. Listeriosis mainly affects susceptible populations including pregnant women and newborns, elderly and immunocompromised individuals. Therefore, detailed analysis of the impact of age (especially early life and old age) and specific factors (such as sex, pregnancy, immunosuppression…) on the effectiveness of trained immunity will be critical to optimize interventions based on trained immunity (57, 58). Moreover, the protection afforded by trained immunity should be tested using different models of bacterial, fungal and viral infections, and with different training agents.

In summary, the induction of trained immunity durably protected from systemic listeriosis by increasing myelopoiesis and the antimicrobial response of mature innate immune cells. Further studies will be required to delineate the persistence of trained immunity. This will give information necessary to consider the possible influence of trained immunity on the development of inflammatory and age-associated diseases and the development of trained-immunity based strategies to enhance or inhibit innate immune responses.

## Data Availability Statement

The raw data supporting the conclusions of this article will be made available by the authors, without undue reservation.

## Ethics Statement

The animal study was reviewed and approved by Service des Affaires Vétérinaires, Direction Générale de l’Agriculture, de la Viticulture et des Affaires Vétérinaires (DGAV), état de Vaud (Epalinges, Switzerland).

## Author Contributions

TR conceived the project. CT, MR, TH, IS, NA, NM, FP, and DLR designed and performed experiments. All the authors analyzed the data, prepared figures and text. TR wrote the paper. All authors contributed to the article and approved the submitted version.

## Funding

TR is supported by the Swiss National Science Foundation (SNSF, grant number 320030_149511 and 310030_173123), by Fondation Carigest/Promex Stiftung für die Forschung (Genève, Switzerland), Fondation Biochimie (Epalinges, Switzerland) and the European Sepsis Academy Horizon 2020 Marie Skłodowska-Curie Action: Innovative Training Network (MSCA-ESA-ITN, grant number 676129, support to CT and IS). TH, CT, and IS received a scholarship from the Société Académique Vaudoise (Lausanne, Switzerland), and NA from the Porphyrogenis Foundation (Lausanne, Switzerland).

## Conflict of Interest

The authors declare that the research was conducted in the absence of any commercial or financial relationships that could be construed as a potential conflict of interest.

## Publisher’s Note

All claims expressed in this article are solely those of the authors and do not necessarily represent those of their affiliated organizations, or those of the publisher, the editors and the reviewers. Any product that may be evaluated in this article, or claim that may be made by its manufacturer, is not guaranteed or endorsed by the publisher.
