# Supplementary material for: Trained Immunity Confers Prolonged Protection From Listeriosis
Source: Front Immunol. 2021 Sep 17;12:723393. doi: 10.3389/fimmu.2021.723393 (PMC8484647; doi:10.3389/fimmu.2021.723393)
Supplement: Supplementary file 1 [file DataSheet_1.docx]

**Supplementary Information**

**Reagents**

| **Reagent** | **Source** | **Identifier** |
| --- | --- | --- |
| 2-deoxyglucose | Sigma-Aldrich | D6134 |
| 5x RIPA Buffer IV with Triton-X-100 (pH 7.4) | Bio Basic | RB4478 |
| Antimycin A from *Streptomyces sp.* | Sigma-Aldrich | A8674 |
| Bovine serum albumin | Sigma-Aldrich | A7906 |
| Brain heart infusion | Oxoid | CM1135 |
| Carbonyl cyanide-p-trifluoromethoxyphenylhydrazone (FCCP) | Sigma-Aldrich | C2910 |
| Celltak | Corning | 354240 |
| Columbia III Agar with 5% Sheep Blood | BD | 254098 |
| cOmplete, Mini, EDTA-free Protease Inhibitor Cocktail | Roche | **11836170001** |
| CpG: G*GGTCAACGTTGAG*G*G*G*G*G | Microsynth |  |
| Fetal bovine serum | Biochrom AG (Merck) | S 0615 |
| HBSS | ThermoFisher | 14175 |
| HBSS no phenol red no Ca+ no Mg+ | ThermoFisher | 88284 |
| IMDM, GlutaMAX | ThermoFisher | 31980-022 |
| KAPA SYBR Green Fast ROX low | Kapa Biosystems | KK4620 |
| LPS ultrapure from *Salmonella minnesota* | List Biologicals | 434 |
| LS columns | Miltenyi Biotec | 130-042-401 |
| Luminol sodium salt | Carbosynth | FL02733 |
| Monocyte Isolation Kit (BM), mouse | Miltenyi Biotec | 130-100-629 |
| Mouse Custom ProcartaPlex | Invitrogen | PPX-MX7DPRR |
| Mouse IL-12p40 DuoSet ELISA | R&D Systems | DY499 |
| Mouse IL-1b Elisa Ready-SET-Go! | eBioscience | 88-7013-88 |
| Mouse IL-6 DuoSet ELISA | R&D Systems | DY406 |
| Mouse Myeloperoxidase DuoSet ELISA | R&D Systems | DY3667 |
| Mouse TNF-α DuoSet ELISA | R&D Systems | DY410 |
| Neutrophil Isolation Kit, mouse | Miltenyi Biotec | 130-097-658 |
| Oligomycin from *Streptomyces* | Sigma-Aldrich | A4876 |
| gentleMACS™ C Tubes | Miltenyi Biotec | 130-093-237 |
| Pam_3_CSK_4_ | EMC microcollections | L2000 |
| Penicillin-streptomycin | ThermoFisher | 15140-122 |
| Phytohemagglutinin, M form (PHA) | ThermoFisher | 10576015 |
| QuantiTect reverse transcription kit | Qiagen | 205313 |
| RNeasy kit | Qiagen | 74106 |
| Rotenone | Sigma-Aldrich | R8875 |
| RPMI 1640 Medium, GlutaMAX | ThermoFisher | 61870-010 |
| Seahorse XF DMEM Medium, pH 7.4 | Agilent | 103575 |
| Seahorse XFp Fluk Pak | Agilent | 103025 |
| Sodium Bicarbonate solution (7.5%) | Sigma-Aldrich | S8761 |
| Recombinant Murine MIP-2 (CXCL2) | Preprotech | 250-15 |
| Costar Transwell®, 6.5 mm, TC treated, 5.0 μm pore size, 24-well cluster plate with lid | Corning | 3421 |
| XF 1.0 M Glucose solution | Agilent | 103577 |
| XF 100 mM Pyruvate solution | Agilent | 103578 |
| XF 200 mM Glutamine solution | Agilent | 103579 |

**Mouse strain**

| **Name** | **Source** | **Identifier** |
| --- | --- | --- |
| C57BL/6J mice | Charles River | 632 |

**Microorganisms**

| **Species** | **Strain** | **Reference** |
| --- | --- | --- |
| *C. albicans* | 5102 |  |
| *L. monocytogenes* | 10403s | NCBI:txid393133 |

**Antibodies and viability dyes used for flow cytometry**

| **Target** | **Clone name** | **Coupling** | **Brand** | **Reference** |
| --- | --- | --- | --- | --- |
| CD3e | 17A2 | APC | eBioscience | 17-0032 |
| CD3e | 17A2 | PE-Cy7 | BioLegend | 100220 |
| CD4 | GK1.5 | APC-Cy7 | BioLegend | 100414 |
| CD8a | 53-6.7 | FITC | BioLegend | 100706 |
| CD11b | M1/70 | APC-eFluor780 | eBioscience | 47-0112 |
| CD11b | M1/70 | PE-Cy7 | BioLegend | 101216 |
| CD11b | M1/70 | PercCP-Cy5.5 | eBioscience | 45-0112 |
| CD11c | N418 | APC | BioLegend | 117310 |
| CD11c | N418 | PE | BioLegend | 117307 |
| CD19 | 1D3 | PE-Cy7 | eBioscience | 25-0193 |
| CD41 | MWReg30 | eFluor450 | eBioscience | 48-0411 |
| CD45 | 30-F11 | FITC | BD Biosciences | 553079 |
| CD45R/B220 | RA3-6B2 | PE-Cy7 | BioLegend | 103222 |
| CD45R/B220 | RA-6B2 | Brilliant Violet 570 | BioLegend | 103237 |
| CD48 | HM48-1 | APC-Cy7 | BioLegend | 103432 |
| CD117 | 2B8 | APC | BioLegend | 105812 |
| CD135/Flt3 | A2F10 | PE | eBioscience | 12-1351 |
| CD150 | TC15-12F12.2 | PerCP-Cy5.5 | BioLegend | 115922 |
| Fixable Aqua | - | - | ThermoFisher | L34957 |
| Fixable Violet | - | - | ThermoFisher | L34955 |
| Ly6C | HK1.4 | PerCP-Cy5.5 | eBioscience | 45-5932 |
| Ly6C/G | RB6-8C5 | PE-Cy7 | BioLegend | 108416 |
| Ly6G | 1A8 | eFluor450 | eBioscience | 48-9668 |
| Ly-76 | TER119 | PE-Cy7 | BioLegend | 116222 |
| MHCII | M5/114.15.2 | PE | eBioscience | 12-5321 |
| Sca-1 | D7 | FITC | eBioscience | 11-5981 |
| Zombie | - | - | BioLegend | 77168 |

**Primers used fot RT-PCR**

| Primer | Sequence (5’-3’) |
| --- | --- |
| *Actin* Forward | CGCAAAGACCTGTATGCCAAT |
| *Actin* Reverse | GGGCTGTGATCTCCTTCTGC |
| *Mpo* Forward | CAGGACGTGAGGGTGACATG |
| *Mpo* Reverse | GCTTCGTCTGTTGCAGTG |
