# Supplementary figures and images for: Trained Immunity Confers Prolonged Protection From Listeriosis
Source: Front Immunol. 2021 Sep 17;12:723393. doi: 10.3389/fimmu.2021.723393 (PMC8484647; doi:10.3389/fimmu.2021.723393)

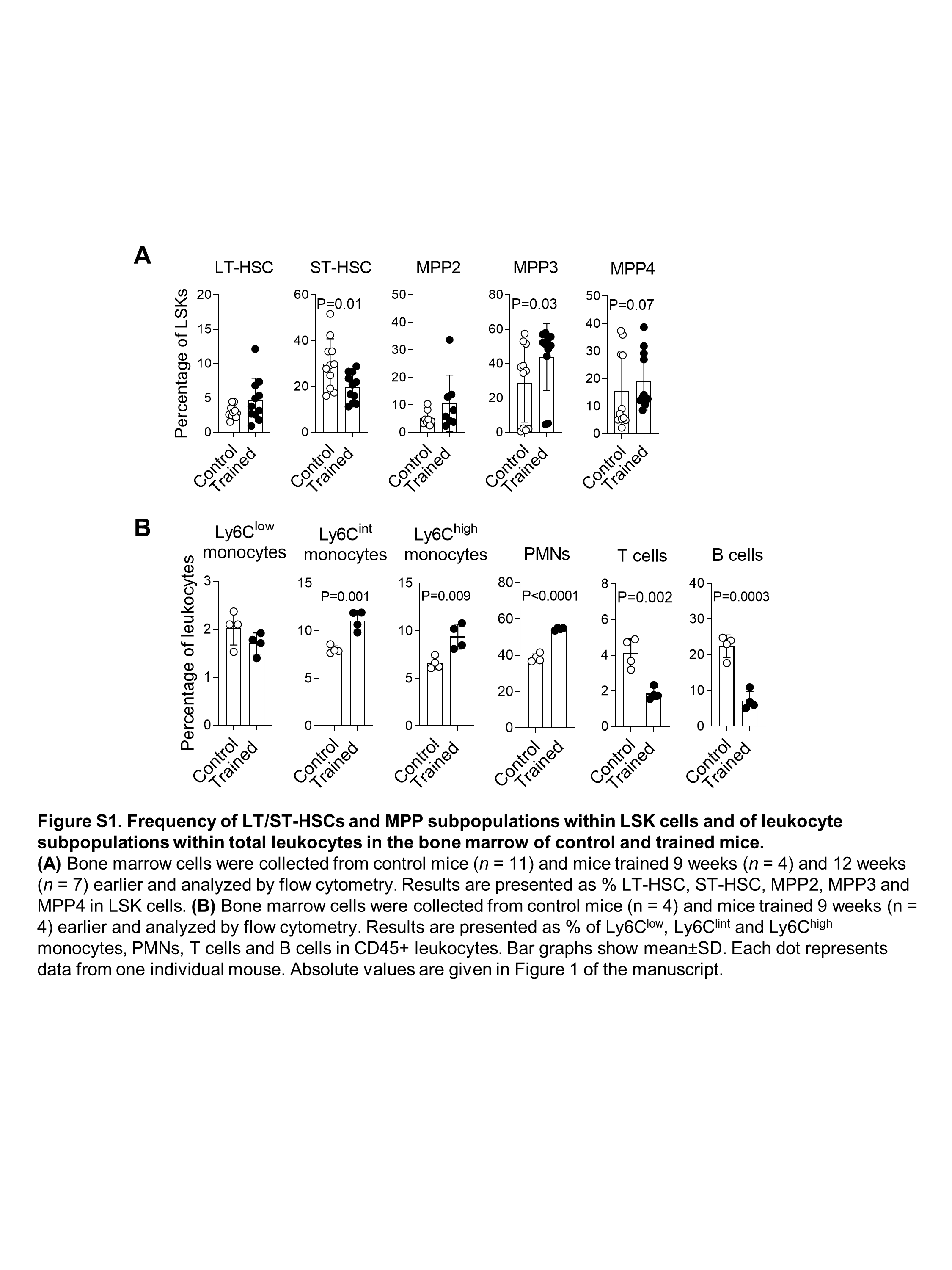

Supplement: Supplementary file 2 [file Image_1.tif]

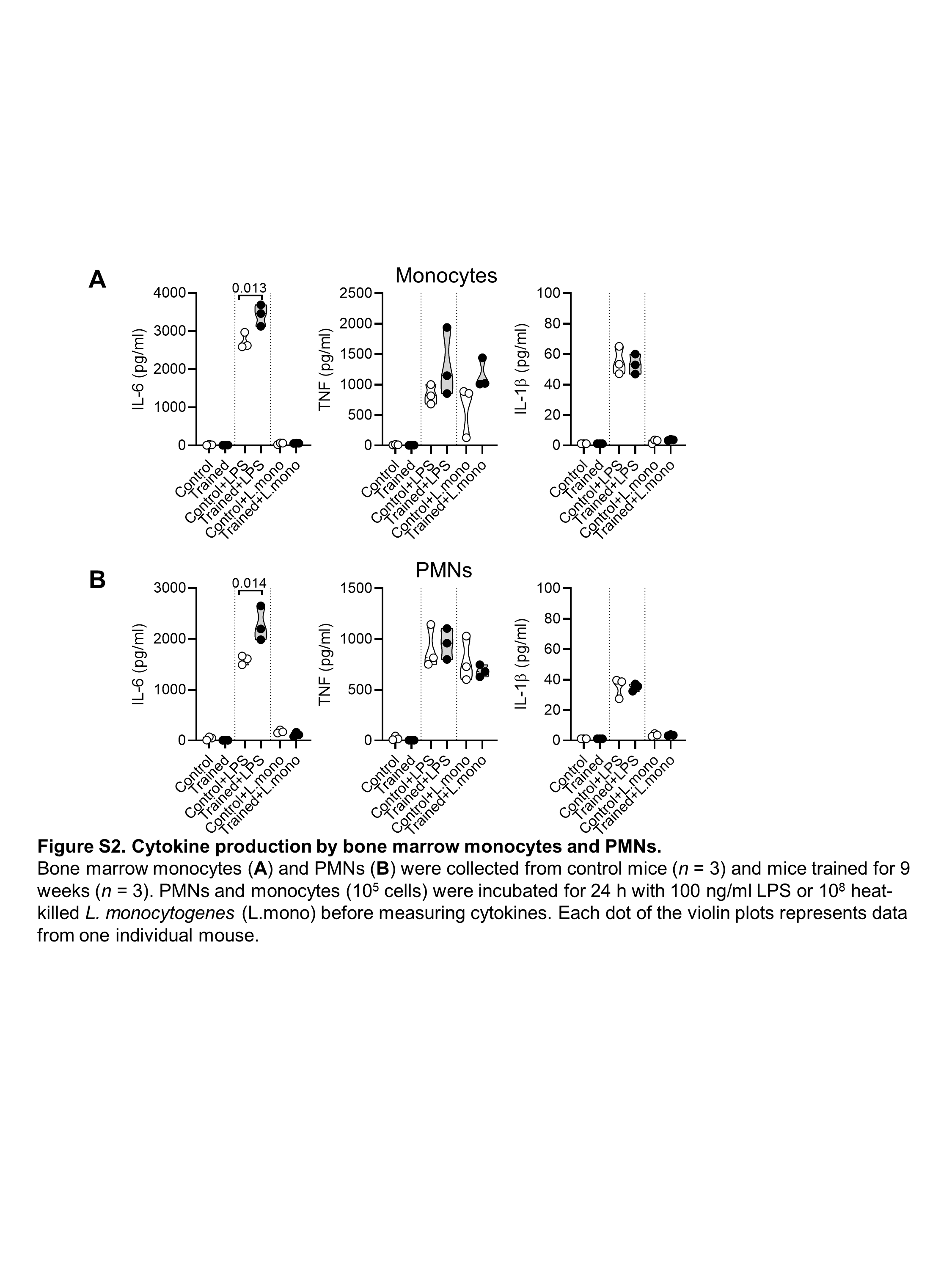

Supplement: Supplementary file 3 [file Image_2.tif]
